# Predicting Early Disease Recurrence of Pancreatic Cancer following Surgery: Determining the Role of NUDT15 as a Prognostic Biomarker

**DOI:** 10.3390/curroncol29040206

**Published:** 2022-04-06

**Authors:** Daniel Llwyd Hughes, Frances Willenbrock, Zahir Soonawalla, Somnath Mukherjee, Eric O’Neill

**Affiliations:** 1 Department of Oncology, University of Oxford, Oxford OX3 7DQ, UK; f.willenbrock@hotmail.co.uk (F.W.); somnath.mukherjee@oncology.ox.ac.uk (S.M.); eric.oneill@oncology.ox.ac.uk (E.O.); 2 Department of Hepatobiliary and Pancreatic Surgery, Oxford University Hospitals NHS, Oxford OX3 7LE, UK; zahir.soonawalla@ouh.nhs.uk

**Keywords:** pancreatic cancer, early recurrence, survival, biomarker, prognosis, NUDT15

## Abstract

**Simple Summary:**

Overall survival rates for pancreatic cancer remain poor. Surgery serves as the only curative treatment strategy. A proportion of patients develop early disease recurrence despite undergoing adjuvant chemotherapy. The ability to identify this high-risk patient cohort may allow for a personalised treatment strategy in terms of both the timing (i.e., neo-adjuvant versus adjuvant) and the regime of choice for chemotherapy, in addition to potentially determining the appropriate intensity of clinical and imaging surveillance following surgery. This study identified NUDT15 expression as a promising biomarker that can identify patients who recur with a relapsing disease within 12 months of their surgery.

**Abstract:**

Surgical resection remains the only curative treatment strategy for Pancreatic Ductal Adenocarcinoma (PDAC). A proportion of patients succumb to early disease recurrence post-operatively despite receiving adjuvant chemotherapy. The ability to identify these high-risk individuals at their initial diagnosis, prior to surgery, could potentially alter their treatment algorithm. This unique patient cohort may benefit from neo-adjuvant chemotherapy, even in the context of resectable disease, as this may secure systemic control over their disease burden. It may also improve patient selection for surgery. Using the Cancer Genome Atlas dataset, we first confirmed the poor overall survival associated with early disease recurrence (*p* < 0.0001). The transcriptomic profiles of these tumours were analysed, and we identified key aberrant signalling pathways involved in early disease relapse; downregulation across several immune signalling pathways was noted. Differentially expressed genes that could serve as biomarkers were identified (BPI, C6orf58, CD177, MCM7 and NUDT15). Receiver operating characteristic curves were constructed in order to identify biomarkers with a high diagnostic ability to identify patients who developed early disease recurrence. NUDT15 expression had the highest discriminatory capability as a biomarker (AUC 80.8%). Its expression was confirmed and validated in an independent cohort of patients with resected PDAC (*n* = 13). Patients who developed an early recurrence had a statistically higher tumour expression of NUDT15 when compared to patients who did not recur early (*p* < 0.01). Our results suggest that NUDT15 can be used as a prognostic biomarker that can stratify patients according to their risk of developing early disease recurrence.

## 1. Introduction

Treatment failure in PDAC management is due to either a loco-regional or a metastatic recurrence [1]. The ESPAC 4 trial was a multicentre randomised adjuvant therapy trial where patients with resectable PDAC were randomised to receive either gemcitabine monotherapy or gemcitabine and capecitabine combination therapy. It illustrated that of the 730 included patients, 65.6% suffered from a disease recurrence during follow-up [2]. A local recurrence was the most frequent recurrence pattern (49.7% of all recurrences), closely followed by distant metastatic recurrence in 40.3% of patients [2]. The time to disease recurrence varied between sites; however, distant sites recurred more quickly when compared to loco-regional recurrence [2]. Early recurrence of PDAC following surgery is a well-described pathological phenomenon [3]. Groot et al., 2018 reviewed a single centre’s experience of 692 patients who underwent a pancreatectomy for PDAC [1]. They noted that liver-only recurrence occurred quickly during follow-up: strikingly, at a median of 6.9 months post-operative [1]. Given the speed of these recurrences, it is highly likely that these patients had micro-metastatic disease at their initial diagnosis; this metastatic disease burden was not detectable on routine cross-sectional imaging techniques.

The ability to identify these individuals with aggressive tumour biology and a high risk of early relapse at their initial diagnosis provides a unique opportunity for delivering precision therapy, specifically in relation to the timing of treatment. For this high-risk patient cohort, the conventional treatment algorithm (surgery followed by adjuvant therapy) may be incorrect. In light of their high risk of disease relapse, they may benefit from upfront systemic treatment prior to surgery, even in the context of resectable disease. Not only would it allow early systemic control over the disease, but it would also provide an opportunity both to study the biology of the tumour and also to improve the patient selection process for surgery. If patients show disease progression whilst on systemic therapy, this is clearly indicative of highly unfavourable tumour biology, and resection should not be pursued [4]. The ability to identify individuals at higher risk of early disease recurrence could also influence and alter the infrastructure of follow-up after surgery, leading to more frequent surveillance. To date, the evidence base for neoadjuvant therapy for PDAC is predominantly centred on patients with locally advanced or borderline-resectable tumours [5,6]. Its role in resectable tumours has yet to be determined. Conflicting data have been published on its use in this specific patient cohort (5). It is possible that the benefit of neoadjuvant therapy in resectable PDAC has yet to be determined due to the heterogeneity of the patient cohort. The ability to risk-stratify patients on the basis of their likely timing of recurrence may identify which patients should complete systemic therapy prior to resection. As such, precision therapy may be delivered by identifying which patients should receive an alternative treatment strategy. This study aims to identify novel biomarkers that are predictive of early disease recurrence.

## 2. Materials and Methods

### 2.1. Establishing a Patient Cohort with Resected PDAC

This study was approved by the Oxford Radcliffe Biobank. Ethical approval was obtained for the acquisition of human tumour tissue samples (Biobank reference 19/A056). All patients recruited to the study provided written consent confirming their voluntary participation and permission for tissue donation for research. Patients were identified from the weekly Hepato-Pancreato-Biliary (HPB) Multidisciplinary Team (MDT) meeting. The inclusion criteria for this study were any patient >18 undergoing elective surgery for PDAC. All patients underwent a full-body staging computerized tomography (CT) and a PET scan to assess for oligometastatic disease. We utilised the NCCN criteria to define resectable disease [7]. Our institutional practice with neo-adjuvant chemotherapy is that it is reserved for patients with borderline or locally advanced disease. After surgical resection of the tumour, a pathologist processed the specimen, and a 5 mm punch biopsy of the centre of the tumour was obtained. All patients were followed up prospectively, and clinical data regarding adjuvant treatment, survival outcomes and recurrence rates were collected.

### 2.2. Histopathological Processing and Immunohistochemical (IHC) Staining

All tumour samples were placed in 5 mL of 10% neutral buffered formalin for 12 h after acquisition. This was followed by a 24 h incubation in 70% neutral buffered formalin. Tumour samples were processed using an STP 120 Spin Tissue Processor (ThermoScientific, Waltha, MA, USA). Embedding in paraffin wax was performed using a HistoStar tissue embedder (Thermo). A microtome (Leica RM 212S, Wetzlar, Germany) was used to cut and create 4 μm sections that were mounted onto positively charged glass slides.

For the histological analysis of the Formalin Fixed Paraffin Embedded (FFPE) sections of the PDAC tumours, all slides were initially de-waxed in 2 separate Citroclear washes for 2 min each. The FFPE slides were then gradually rehydrated in a decreasing concentration of ethanol (a total of 5 concentrations were used: 100%, 100%, 90%, 70% and 50%) for 3 min each. The slides were subsequently washed in running tap water for 5 min. Selective staining of the slides was performed for Hematoxylin and Eosin (H&E) and Immunohistochemistry (IHC). After staining, all slides were gradually dehydrated in ethanol (the ethanol concentrations used were 50%, 70%, 90%, 100% and 100%) for 30 s each. Excess ethanol was removed by performing 2 washes for 1 min in Xylene. Slides were mounted with coverslips using Di-N-Butyl Phthalate in Xylene (DPX). Slides were left to dry overnight in the chemical fume hood. The Aperio Scanscope slide scanner was used to scan slides and to create digitalized pathology images.

IHC was performed for NUDT15 protein expression within the tumours. The conditions for the antibody were optimised with respect to antigen retrieval method, retrieval buffer and antibody dilution. The retrieval buffer used for antigen retrievals was a Citrate Buffer (pH6) with 0.05% Tween20. A heat-induced antigen retrieval method was used by placing a portable pressure cooker (Nordic ware, Minneapolis, MN, USA) within a microwaving and heating the samples on full power for 13 min. Samples were left to cool at room temperature for 30 min. The Atlas (HPA038969) antibody was used to stain for the NUDT15 protein at a concentration of 1:600. All slides were incubated overnight in a humid chamber at 4 °C with the primary antibody. A secondary antibody conjugated with HRP was used to label the slides. Under direct microscopic vision, immunodetection was undertaken using a DAB substrate kit (Agilent Dako, Santa Clara, CA, USA). Digital images were obtained on the Aperio Slide scanner Software, Imagescope 12.3.3 (Leica Biosystems, Wetzlar, Germany). The quantification of IHC staining was performed using ImageScope and ImageJ. Tumour ROIs were selected and exported as JPEG images. The image was converted into an 8-bit image prior to selecting a threshold that created a mask over the positive DAB reaction. This mask was subsequently measured and was used to calculate the % positive area within the ROI.

### 2.3. Analysis of RNA Sequencing Data

Bioinformatics analysis was performed on the Cancer Genome Atlas (TCGA) Pancreatic Adenocarcinoma (PAAD) dataset. The RNA sequencing data and the corresponding patient clinical information (version 2016 1 28) were downloaded through the Firebrowse portal (http://firebrowse.org/) (accessed on 6 October 2021). The ‘dplyr’ package was used for data ‘cleaning’ and filtering [8]. The analysis of RNA sequencing data was only performed on the TCGA dataset.

The differential expression was calculated using two separate packages (edgeR and DESeq2) in order to improve the robustness of the results [9,10]. Data generated from the edgeR analysis were used for creating graphical plots. The patient cohort with early recurrence was defined as the population of interest for the purpose of differential gene expression analysis, whereas the remaining cohort (no early recurrence) served as the control population. After the visualization of expression data, the threshold for inclusion was set at genes with a read count < 1 count per million (CPM) in at least 9 samples. The trimmed mean method (TMM) was used within edgeR to determine the normalization factors for the respective gene library sizes. Differences in gene expressions were calculated and incorporated into a negative binomial model. A False Discovery Rate (FDR) of ≤ 0.01 was used as a threshold for gene identification.

Gene set enrichment analysis (GSEA) was performed using the ‘fgsea’ package (3.14) [11]. Predefined gene sets of the ‘hallmarks of cancer’ were downloaded from https://www.gsea-msigdb.org/gsea/index.jsp (accessed on 6 October 2021). KEGG pathways were downloaded from the Kyoto Encyclopedia of Genes and Genomes online: https://www.genome.jp/kegg/ (accessed on 6 October 2021). The edgeR results were compared to the ranked gene list. Significant pathway enrichment was defined by an FDR < 0.01. GSEA pathway analysis results were presented as stacked bar-charts.

Validation of biomarkers for their diagnostic ability to identify early recurrence was performed by computing generalized linear models. Receiver Operating Characteristic (ROC) curves were calculated and visualised using the ‘pROC’ package (1.18) [12]. The area under the ROC curve (AUC) was calculated for each biomarker. An AUC value < 0.5 was considered as a diagnostic failure with an inability to differentiate between the two desired patient cohorts.

### 2.4. Statistical Analysis

All statistical analysis was performed in R version 3.6.3 [13]. Survival analysis was calculated using the Survival package in R [14]. Cox Proportional Hazard Regressions were calculated and visualized with Kaplan-Meier curves. Data analysis consisted of initially assessing the distribution of the data. In order to determine whether the data were normally distributed, the Shapiro–Wilk test was performed. Numerical data that were parametric were assessed with an unpaired Student’s *t*-test, whereas non-parametric data were assessed with a Mann-Whitney U test. A comparison of means across multiple independent groups was performed with a one-way ANOVA with Tukey’s post hoc adjustment. For the analysis of categorical variables, a Chi-squared independence test was performed, unless a sample size of <10 was present, in which case a Fischer’s exact test was performed. Data from IHC staining were presented as mean ± SEM (error bars). All statistical results <0.05 were deemed statistically significant.

## 3. Results

**Figure 1:**
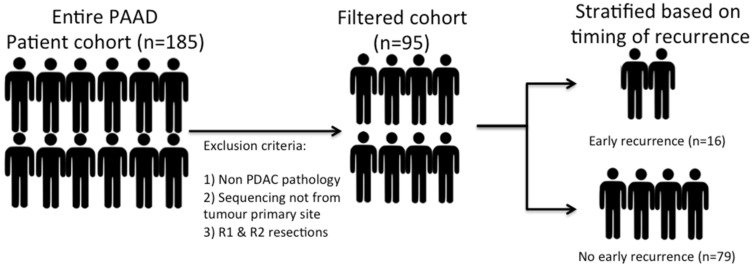
Overview of patient selection process from the TCGA dataset. The initial patient cohort was highly heterogeneous and was not uniform in nature. Filtering and exclusion of cases were performed based on the following criteria: non-PDAC pathology, sequencing data derived not from the primary tumour and any patient who had a positive surgical resection margin (R1 or R2 status). Patients were ultimately stratified based on the timing of their recurrence into two cohorts: early recurrence and the comparator cohort of no early recurrence.

RNA sequencing data and accompanying clinical data were obtained from the online TCGA data archive for PDAC. A total of 185 patients are included within this dataset. However, a highly heterogeneous patient population was noted, and as such, patients with non-PDAC pathology (neuroendocrine tumours or rare subtypes of PDAC) and patients who had sequencing performed from non-primary tumour sites (e.g., biopsied liver metastases or peritoneal disease) were excluded in order to create a uniform patient cohort. Patients with an R1 or R2 resection margin status were also excluded from the analysis. The presence of a positive margin could impact the interpretation of local recurrence rates. Excluding the patients with a positive margin created a smaller but homogenous patient cohort, all of whom had undergone a curative resection. This resulted in a final cohort of 95 patients (Figure 1). A 12-month post-operative time point was used as a reference point to define the timing of a recurrence. The cohort was subsequently divided into two groups: “early recurrence” and “no early recurrence”.

**Figure 2:**
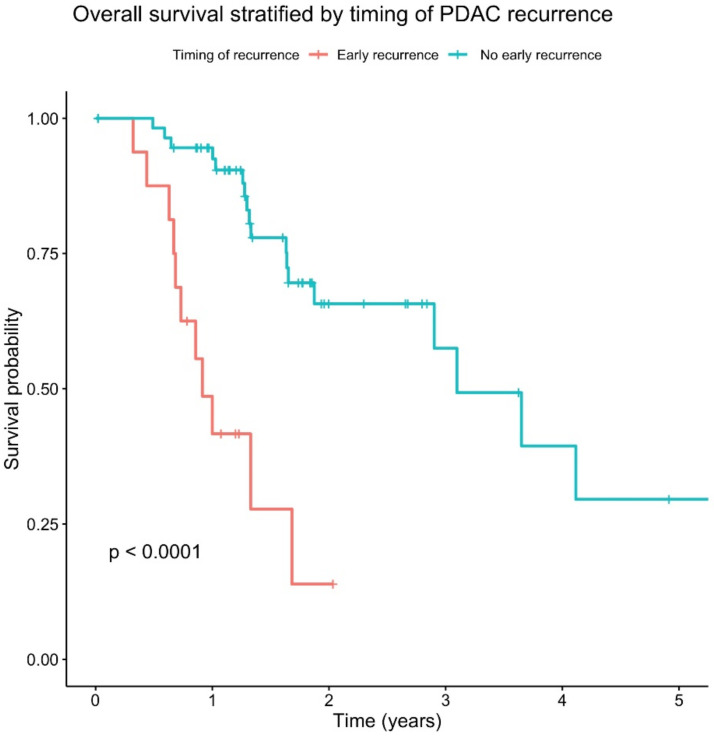
Impact of early recurrence on overall survival. Kaplan-Meier survival curve of overall survival trends of PDAC patients stratified by timing of recurrence. The patient cohort with an early recurrence (<12 months) following resection had a statistically significant reduced overall survival (*p* < 0.0001).

Firstly, in order to determine whether early disease recurrence has a clinically meaningful impact on patient survival, a survival analysis was performed on the cohort. Early disease recurrence was associated with a significantly worse overall survival (*p* < 0.0001) (Figure 2).

In light of the significant impact on survival, we investigated whether there was any difference within the patient demographics or the histopathological features of these tumours. There was no statistical difference between the demographics of either cohort or the aetiological risk factors of PDAC (Table 1). Interestingly, there was also no statistical difference in the gross histopathological features between these tumours. Therefore, patients who recurred early did not have larger or more advanced (as per staging) tumours when compared to patients who did not have an early recurrence, but this may have been due to the small sample size.

**Figure 3:**
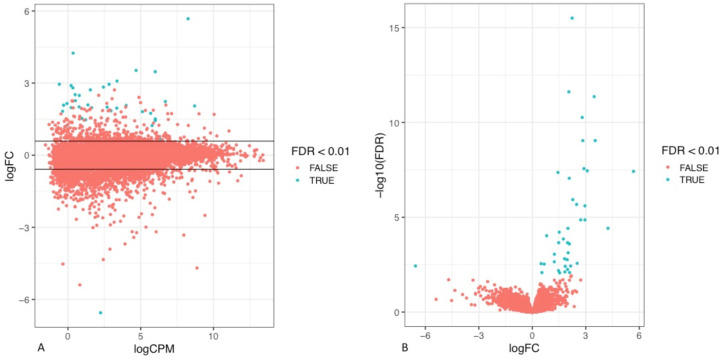
Identification of differentially expressed genes in patients with an early disease recurrence. (**A**) Smear plot illustrating the relationship between gene expression (logCPM) and the log FC of differentially expressed genes. An absolute fold change of 1.5 is represented by the horizontal threshold lines. (**B**) Volcano plot illustrating the logFC of the differentially expressed genes against the negative log10FDR in order to present the most significant results.

To assess the differences in the transcriptomic profile of tumours that recurred early in comparison to those that did not, the RNA sequencing data (RNA seq) of the tumours of the respective cohorts were analysed. Through visual exploration of the sequencing data, it was apparent that a statistically significant difference in the gene expression profile of a number of genes was present between the two patient cohorts defined by their timing of disease relapse (Figure 3, Supplementary Table S1).

**Figure 4:**
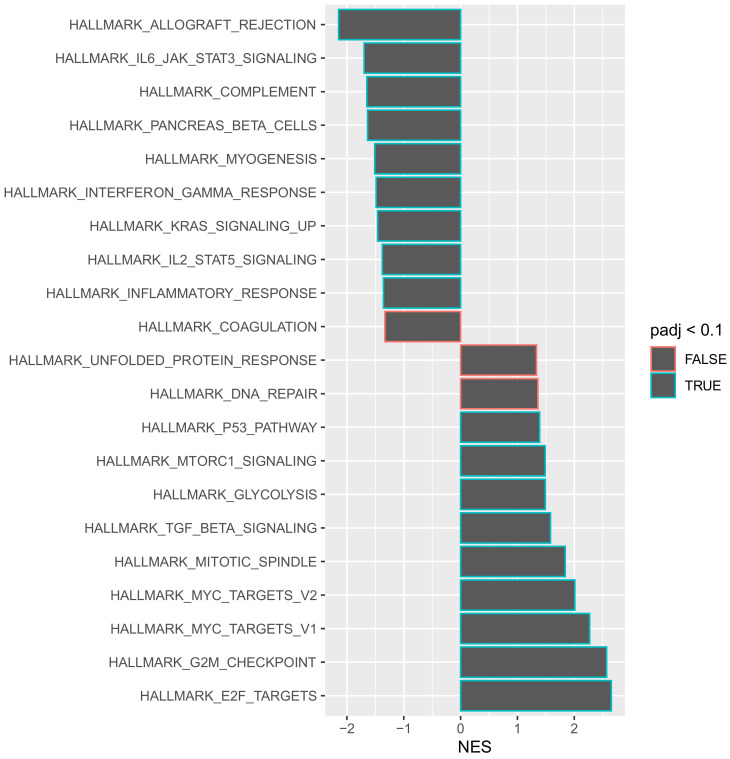
Pathway enrichment analysis of the normalised enrichment scores (NES) of the 50 hallmark cancer pathways presented in a stacked bar chart ordered by FDR. A total of 18 different cancer pathways were enriched within tumours that recurred with 12 months of resection. FDR—False Discovery Rate.

Gene set enrichment analysis allows for genes that share a common functionality to be grouped together within a specific pathway for analysis. Such an approach provides an opportunity to obtain a global view of how individual genes may act in synergy to change the behaviour of a tumour. To understand the aggressive disease phenotype present in the tumours that recurred early, a pathway enrichment analysis was performed on the predefined hallmarks of cancer pathways using a false discovery rate (FDR) of 0.1. Of the 50 hallmarks of cancer pathways analysed, 18 were significantly enriched at the 0.1 FDR cut-off value (Figure 4).

An interesting observation is that the most significantly downregulated pathway was “HALLMARK_ALLOGRAFT_REJECTION”. This pathway includes numerous genes that are routinely upregulated during transplant graft rejection [15]. The selective downregulation of this pathway suggests that there is a generalised immune suppressive function within these tumours that recur early. In order to evaluate whether a specific immune pathway was involved, a further gene set enrichment analysis was performed using the Kyoto Encyclopaedia of Genes and Genomes (KEGG) pathways database. The KEGG database is a comprehensive archive of several different pathways that is not solely restricted to hallmarks-of-cancer-related pathways. It was noted that 34 different KEGG pathways were significantly downregulated within tumours that recurred within 12 months of resection (Supplementary Figure S1). These included several independent immune function pathways such as T cell receptor signalling, B cell receptor signalling, chemokine signalling, toll-like receptor signalling and natural killer cell mediated cytotoxicity (Supplementary Material). The numerous downregulated independent immune pathways suggest that these tumours promote an immune-suppressive environment and may therefore utilise immune escape as a mechanism to avoid detection after systemic dissemination and subsequently relapse early.

**Figure 5:**
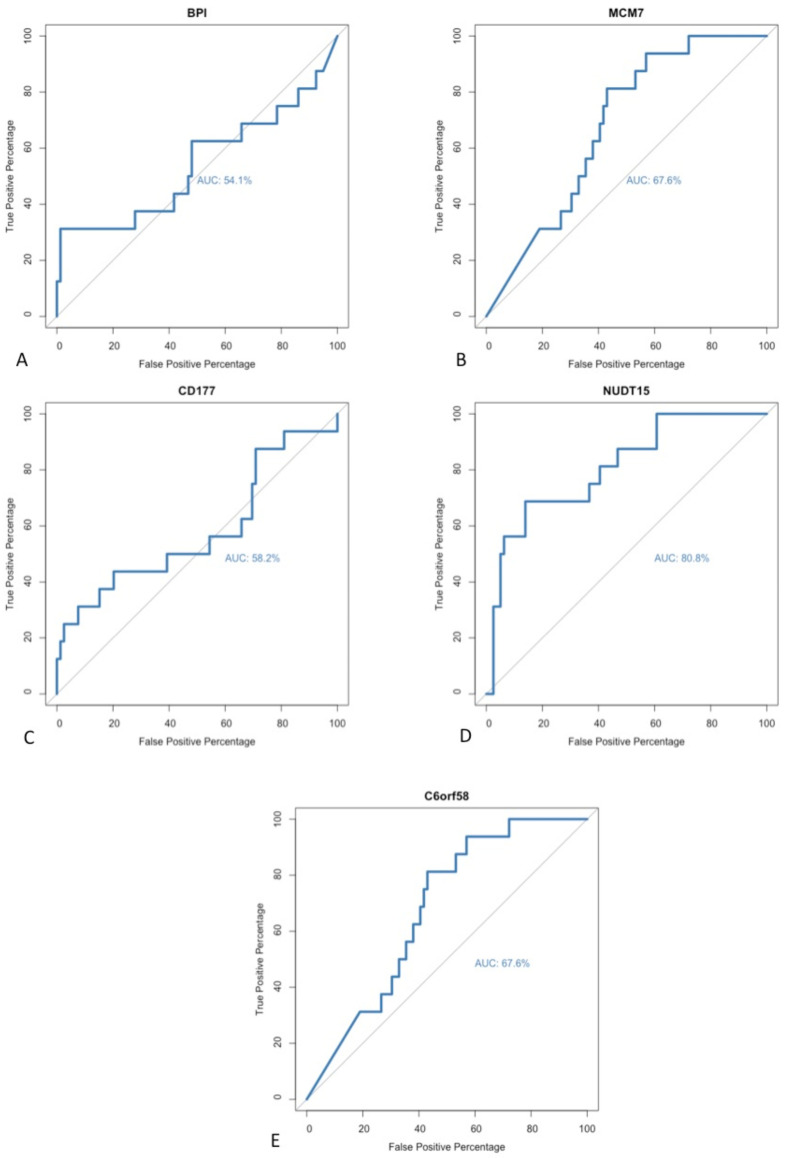
Receiver operating characteristic curve analysis to determine biomarker sensitivity. ROC curves and area under the curve (AUC) calculated for (**A**) BPI, (**B**) MCM7, (**C**) CD177, (**D**) NUDT15 and (**E**) C6orf58.

No universal method has been accepted as a gold standard technique for identifying biomarkers during exploratory analyses of sequencing data. DE gene analysis was performed using two independent software packages. A paired matching algorithm was run in order to identify individual differentially expressed genes that had been identified as statistically significant within both software packages. A positive correlation was noted between the genes identified in both algorithms. A total of five different genes were identified as significant within both software programmes; these genes included BPI, C6orf58, CD177, MCM7 and NUDT15. In order to further evaluate these potential biomarkers for detecting early recurrence, a repeated receiver operating characteristic (ROC) curve analysis was performed to determine their respective sensitivities and diagnostic abilities. The area under the curve (AUC) was calculated for each ROC curve in order to assess the model’s performance (Figure 5). All five of the identified targets had an AUC value >50%. However, within the field of biomarker discovery, the wider research literature suggests that only biomarkers with AUC values >80% should be selected because higher AUC scores are reflective of a superior model with greater diagnostic ability and clinical relevance [16]. As such, only one of the previously described biomarkers met the threshold: NUDT15.

**Figure 6:**
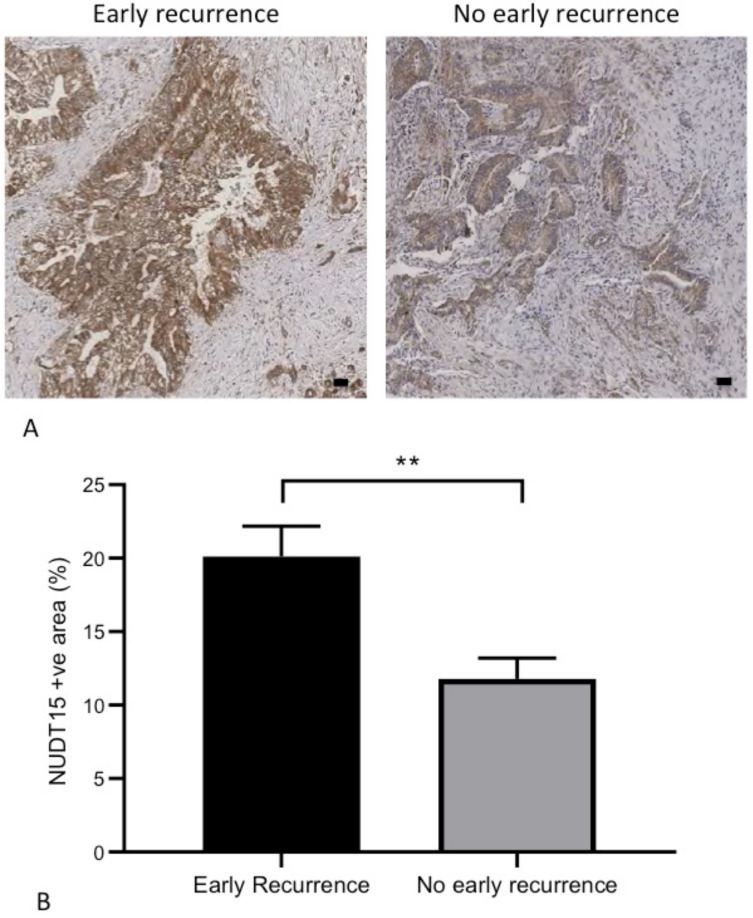
Higher NUDT15 expression is noted within tumours that recur within 12 months of resection. (**A**) Representative IHC staining of NUDT15 expression within patient tumours stratified based of the timing of disease recurrence. Images taken at ×5 magnification, scale bar 50 um. (**B**) Quantification of NUDT15 expression within tumours. Data presented as mean ± SEM, statistical analysis performed by unpaired Student’s-*t* test. Data information: ** *p* < 0.01.

To validate NUDT15 as a biomarker for early recurrence, we reviewed its expression within our own cohort of patients with resected PDAC. Of the 13 patients recruited, three (23%) developed early disease recurrence within 12 months of their surgery. Distant metastases were the most common site of recurrence (liver or lung) (Table 2). NUDT15 protein expression was identified on IHC. A statistically higher (*p* < 0.01) NUDT15 expression was observed amongst tumours that recurred early when compared to tumours that did not recur within 12 months of resection (Figure 6, Supplementary Material).

## 4. Discussions

Within this study, we investigated whether it was possible to risk-stratify patients based on biomarkers that were predictive of early disease recurrence. Being able to identify this high-risk patient cohort would have a significant clinical impact. These patients may benefit from early, systemic therapy prior to resection in order to treat the micro-metastatic disease, or they may require more frequent, early follow-up post-operatively. As observed within our own patient cohort, early recurrence was present in 23% of all patients. The results of the study suggest that NUDT15 is a promising biomarker that can be detected on a protein level within PDAC tumours.

Nudix hydrolase 15, also referred to as NUDT15, is an enzyme from the Nudix hydrolases superfamily [17]. The enzymatic function of this family is to hydrolyse nucleoside diphosphates into nucleoside monophosphate [17]. The activity of the enzymes is induced by oxidative damage or pathological transversion events during DNA replication. Its role in cancer development or metastatic dissemination has yet to be determined. The enzyme has been thoroughly investigated in the context of therapeutic drug toxicity (predominantly with the thiopurine class of drugs of purine antimetabolites) [18]. Mutations in the NUDT15 gene are associated with the inadequate metabolism of thiopurines and may cause myelotoxicity [18]. In light of the high diagnostic ability (as per the AUC score of 80.8%) and its capacity to identify patients at risk of early disease relapse, we wanted to assess whether the protein expression was sufficient for it to be utilised as a biomarker. This may have clinical implications, especially in the context of a peri-operative biopsy (through Endoscopic Ultrasound biopsy) where patients could be risk-stratified for early relapse at the point of their diagnosis.

In order to validate the findings of the exploratory analysis, a literature search on the identified genes was undertaken. Interestingly, it was noted that three of the identified genes (C6orf58, CD177 and MCM7) had been previously identified as prognostic factors for survival in PDAC. Wang et al., 2018 investigated the impact of tumoural infiltration of neutrophils with CD177 expression and demonstrated that high levels of infiltration were associated with a significant reduction in overall survival (*p* = 0.01) [19]. Liao et al., 2018 performed RNA sequencing of early stage PDAC patients and assessed the relationship between MCM gene expression and overall survival [20]. A total of six MCM genes were studied, and higher expression was noted within the tumours when compared to adjacent healthy tissue [20]. Whilst differences were noted in survival trends when patients were stratified by individual MCM gene expression, the most significant impact on survival was in the context of high MCM4 gene expression [20]. Wu et al., 2011 performed a cox regression analysis in order to identify a gene signature associated with long term survival of PDAC [21]. They identified 12 genes of interest, one of which was C6orf58; however, due to the paucity of literature on the gene at the time of publication, they were unable to describe in detail the function of the gene [21]. From the output of the exploratory analysis, three of the five identified genes have been previously described in the context of PDAC. This reinforced the significance of the results.

Biomarker discovery for PDAC has been a very active field over the last decade. Most efforts revolve around developing biomarkers for early disease detection. The principal argument is that by diagnosing patients early, a greater proportion would be amenable to curative surgery. In addition, our current and only licenced diagnostic biomarker (CA19-9) is not without its limitations, notably its false positivity rate and poor predictive value in asymptomatic patients [22]. With the recent acknowledgment of the molecular subclasses of PDAC, research initiatives are now focussing on developing biomarkers that allow quick identification of PDAC subclasses [23]. This ability would allow early risk stratification of patients at the time of their diagnosis. Obtaining this information would also dictate future treatment strategies. A good example of this is the current phase 2 trial PASS-01 that is actively recruiting patients with metastatic PDAC [24]. This randomised clinical trial will evaluate two different chemotherapy regimes in the context of molecular profiling and biomarker validation [24], notably using GATA6 to discriminate between basal and classical tumours. This is a clear example of harnessing the tumour biology to dictate therapy. Our approach with our biomarker discovery was to focus on identifying a subpopulation of patients who were at risk of early recurrence. NUDT15 was associated with the best ROC and AUC value, and as such was further evaluated with IHC staining for protein level expression. Within our validation cohort, a statistical difference was noted in the expression level between patients with and without an early recurrence. There are limitations to note within this study. The small sample size of our patient cohort limits the accuracy of the validation. In addition, only a small proportion of patients suffered from an early recurrence; this may result in an underpowered analysis. Validating the results in a larger patient cohort from another institution would also strengthen the analysis. We validated NUDT15 protein expression on a core biopsy of the tumour, and further research is required in order to ascertain whether there is sufficient tissue yield from Endoscopic Ultrasound (EUS) and to whether NUDT15 can be detected on EUS biopsy material.

## 5. Conclusions

This study identified NUDT15 as a promising biomarker that can identify patients at the highest risk of early relapse after a curative resection. This unique patient cohort may benefit from an alternative treatment strategy, specifically through altering the timing of systemic therapy, modifying the chemotherapeutic treatment regime, or restructuring their surveillance follow-up with an emphasis on early and frequent cross-sectional imaging. However, further validation in a larger retrospective patient cohort is required. What is clear is that we must acknowledge PDAC as a diverse cohort of tumours, and there is an apparent need to develop alternative stratification strategies for prognostic measures, specifically variables that are based on intrinsic tumour biology to guide therapy or to inform survival probability.

## Figures and Tables

**Table 1:** A comparison between the patient demographics and tumour histopathological features between patients with and without early disease recurrence from the TCGA PAAD dataset.

Demographics	Early Recurrence	No Early Recurrence	*p* Value
	(*n* = 16)	(*n* = 79)	
Age (mean)	66	64	0.5
Female	5	38	0.33
Male	11	41	
Past medical history			
History of Chronic Pancreatitis:			
Yes	14	54	0.6
No	1	8	
History of Diabetes:			
Yes	11	46	1
No	4	19	
Smoking pack year history (Mean)	14.4	24.1	0.3
histopathology			
T stage:			
T1	2	3	0.4
T2	2	10	
T3	12	64	
T4	0	1	
N stage:			
N0	5	25	1
N1	11	54	
Stage:			
Stage Ia	1	3	0.9
Stage Ib	1	5	
Stage Iia	3	14	
Stage Iib	11	53	
Stage III	0	1	
Stage IV	0	2	

**Table 2:** Survival outcomes of recruited patients to the validation cohort after surgical resection.

Patient	Date of Surgery	Adjuvant Therapy	Recurrence	Site of Recurrence	Date of Recurrence	Time to Recurrence (Days)	Early Recurrence	Current Status
1	29/07/19	Yes	Yes	Aortocaval nodes	19/11/20	479	No	Alive
2	14/08/19	Yes	No	n/a	n/a	n/a	n/a	Alive
3	27/08/19	No	No	n/a	n/a	n/a	n/a	Dead (13/05/20)
4	29/08/19	Yes	Yes	Lung and Bone	01/06/20	277	Yes	Dead (27/12/20)
5	02/10/19	Yes	Yes	Liver metastases, Portal and SMA nodes	15/08/20	318	Yes	Dead (23/09/20)
6	17/10/19	No	No	n/a	n/a	n/a	n/a	Dead (5/5/21)
7	28/10/19	Yes	No	n/a	n/a	n/a	n/a	Alive
8	07/11/19	No	Yes	Lung, pleural and mediastinal nodes	29/06/21	600	No	Dead (30/07/21)
9	27/11/19	Yes	No	n/a	n/a	n/a	n/a	Alive
10	10/01/20	Yes	Yes	Liver	25/11/20	320	Yes	Alive
11	17/02/20	No	No	n/a	n/a	n/a	n/a	Alive
12	03/03/20	No	No	n/a	n/a	n/a	n/a	Alive
13	06/05/21	Yes	No	n/a	n/a	n/a	n/a	Alive

n/a – Not applicable.

## Data Availability

Data available in a publicly accessible TCGA repository.

## Supplementary Materials

The following supporting information can be downloaded at: https://www.mdpi.com/article/10.3390/curroncol29040206/s1, Figure S1: KEGG pathways enrichment analysis. The normalised enrichment scores (NES) of each pathway are presented within a stacked bar chart that is ordered by the false discovery rate. A total of 34 different KEGG pathways were significantly down regulated and 11 pathways significantly up regulated. Table S1: Table of the top 21 differentially expressed genes between patients with and without an early recurrence following resection for PDAC. Genes are ordered as per a decreasing FDR rate. (logFC = log fold change; logCPM = log counts per million; FDR = False Discovery rate). Table S2: Histopathological tumour features and NUDT15 expression.
