# Supplementary material for: Predicting Early Disease Recurrence of Pancreatic Cancer following Surgery: Determining the Role of NUDT15 as a Prognostic Biomarker
Source: Curr Oncol. 2022 Apr 6;29(4):2516–29. doi: 10.3390/curroncol29040206 (PMC9032287; doi:10.3390/curroncol29040206)
Supplement: Supplementary file 1 [file curroncol-29-00206-s001.zip › curroncol-1589507-supplementary.pdf]

Supplementary material

Table S1 - Table of the top 21 differentially expressed genes between patients with and without an early recurrence following resection for PDAC. Genes are ordered as per a decreasing FDR rate. (logFC = log fold change; logCPM = log counts per million; FDR = False Discovery rate) .

Genes	logFC	logCPM	LR	P Value	FDR
PGAP3	2.234863421	6.692849271	85.43104973	2.40E-20	3.12E-16
ERBB2	2.048574468	8.702957168	66.3790854	3.72E-16	2.42E-12
C20orf56	3.472741222	5.999955935	64.42943056	1.00E-15	4.34E-12
GRIP2	2.800221839	0.34221273	58.88971646	1.67E-14	5.43E-11
HEPACAM2	3.52979217	4.680186101	52.9183579	3.48E-13	9.04E-10
REP15	2.833085067	2.439755412	52.56329868	4.17E-13	9.04E-10
BPI	2.897046925	0.212890127	45.59065609	1.46E-11	2.71E-08
CD177	3.085022682	3.372787741	44.8040761	2.18E-11	3.54E-08
MUC2	5.677339448	8.25616174	44.43688388	2.63E-11	3.80E-08
C17orf37	1.44088583	5.997701124	43.96865629	3.34E-11	4.34E-08
HES6	2.070971354	3.999807969	42.41237216	7.39E-11	8.75E-08
KLHL32	2.268442251	0.512018374	37.16812282	1.08E-09	1.18E-06
PKD1L2	2.484755825	0.781779596	35.88371452	2.09E-09	2.10E-06
CDX1	2.951638627	2.825814057	35.40002688	2.68E-09	2.50E-06
CCL24	2.716997309	1.53944708	31.92261059	1.60E-08	1.37E-05
MYADML2	2.953404861	-0.592385685	31.82951301	1.68E-08	1.37E-05
KLK12	4.246698942	0.35431791	29.66490429	5.14E-08	3.83E-05
APCDD1L	1.999333334	2.693405844	29.60405642	5.30E-08	3.83E-05
GRB7	1.510253759	6.005227248	28.60095229	8.89E-08	6.09E-05
MCM7	0.804754718	6.798879106	27.66823142	1.44E-07	9.37E-05
HSD11B2	1.743807374	5.683524172	26.80139663	2.25E-07	0.00013973

Table S2 – Histopathological tumour features and NUDT15 expression

Patient	Pathology	Tumour differentiation	T stage	N stage	Lymphatic invasion	Vascular invasion	R Status	Surgical procedure	Early recurrence within 12 months	Mean NUDT15 expression (% positive)
1	PDAC	Poor	T2	N0	L0	V0	R0	Whipple	No	5.7
2	PDAC	Moderate	T2	N1 (1/19)	L0	V1	R1	Whipple	No	8.4
3	PDAC	Moderate	T1	N0	L0	V0	R0	TP	No	7.3
4	PDAC	Poor	T2	N2 (9/18)	L1	V1	R0	Whipple	Yes	19.1
5	PDAC	Moderate	T2	N1 (3/49)	L1	V1	R1	Whipple + PV resection	Yes	25.6
6	PDAC	Moderate	T3	N2 (5/24)	L1	V1	R1	Whipple	No	16.1
7	PDAC	Moderate	T1	N1 (2/32)	L1	V0	R0	TP	No	8.2
8	PDAC	Moderate	T1	N2 (4/19)	L1	V1	R1	TP	No	8.7
9	PDAC	Moderate	T2	N1 (3/38)	L1	V1	R1	Whipple	No	16.5
10	PDAC	Moderate	T2	N2 (5/18)	L1	V0	R0	Whipple	Yes	27.1
11	PDAC	Well	T2	N2 (4/52)	L1	V1	R1	Whipple	No	6.1
12	PDAC	Moderate	T2	N0	L0	V0	R0	Whipple	No	2.5
13	PDAC	Moderate	T2	N1 (2/44)	L1	V1	R0	Whipple	No	14.5

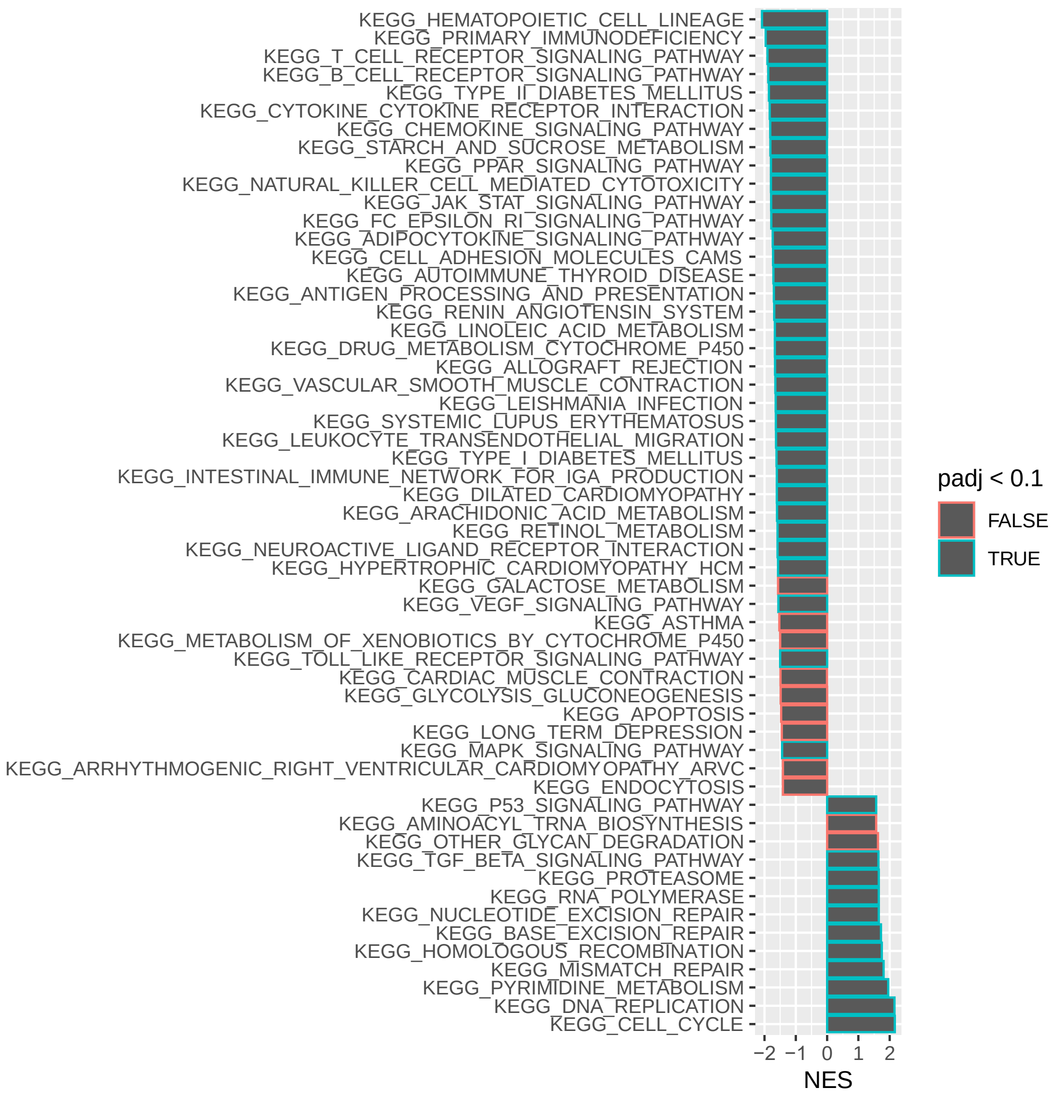

Figure S1 – KEGG pathways enrichment analysis. The normalised enrichment scores (NES) of each pathway are presented within a stacked bar chart that is ordered by the false discovery rate. A total of 34 different KEGG pathways were significantly down regulated and 11 pathways significantly up regulated.
